# Esthesioneuroblastoma: Summary of Single-center Experiences with Focus on Adjuvant Therapy and Overall Survival

**DOI:** 10.7759/cureus.4897

**Published:** 2019-06-13

**Authors:** Hammam A Alotaibi, Stefano M Priola, Anne-Laure Bernat, Faisal Farrash

**Affiliations:** 1 Miscellaneous, Prince Sultan Military Medical City, Riyadh, SAU; 2 Neurosurgery, University of Toronto, Toronto, CAN; 3 Neurosurgery, Lariboisière Hospital, Paris, FRA; 4 Neurosurgery, King Faisal Specialist Hospital and Research Center, Riyadh, SAU

**Keywords:** esthesioneuroblastoma, kadesh score, chemotherapy, enb, overall survival, disease free survival, radiation therapy

## Abstract

Esthesioneuroblastoma (ENB) is a rare malignant tumor of the nasal cavity. The genetic basis of its development is still under study and has not been fully delineated. It has varying symptoms depending on the lesion’s location within the nasal cavity. The most commonly used systems for such lesions are the Kadish staging and Haymes grading systems.

The objectives are to review the most recent published literature evaluating the different treatments/ combination of treatment and assess the most appropriate treatment modality that can provide the longest progression-free survival and overall survival for ENB patients. Moreover, a look at what the latest literature suggests when it comes to adjuvant treatments and their effect on survival is also key to further the body of knowledge for neurosurgeons, Ears, Nose, and Throat (ENT) physicians and all the different subspecialties that deal and serve these population of patients.

The published literature was reviewed starting from 1990. The focus was made on single-center experiences given their availability and easy access. The most recently published systematic review was used as the benchmark; research published after that was included in this study. The database search in OVID was conducted using the following keywords: “Esthesioneuroblastoma”, “ENB”, “Olfactory Neuroblastoma”, Nose neoplasm”, skull base neoplasm”, “radiation”, and “resection”. The database search found 17 papers which included 14 single-center reports, one systematic review, and two nationwide multi-center reviews.

Surgery plus adjuvant radiation therapy appears to provide the best overall survival and progression-free survival especially in patients with high Kadesh grade. On the other hand, surgery alone or biopsy followed with radiation therapy provided the lower progression-free survival and overall survival from time of diagnosis. The role of chemotherapy, however, requires further investigation to assess its potentially harmful effects. The use of surgery as a stand-alone modality of treatment should be cautiously and rarely used in patients with lower staging scores and multiple negative resection margins.

## Introduction and background

Esthesioneuroblastoma (ENB) is a rare malignant tumor of the nasal cavity with distinct clinicopathologic features, multiple facets, and differing clinical behavior [1]. It originates from olfactory cells and also has a neural crest origin. Although it is a rare tumor, it has the potential for aggressive growth and the propensity for regional metastasis [2]. Early discovery and aggressive management play a key role in a patient’s survival and quality of life [3]. ENB was described in 1924, and since then different institutions have developed various protocols for treatment whether it is surgical, radiation, chemo or a combination of these treatment modalities [4]. In modern practice, multimodality/multidisciplinary therapy appears to be the approach of choice [5]. There has not been extensive research on the genetic basis of ENB in recent years but sonic-hedgehog gene has been implicated recently in the development of the tumor. Further investigation is necessary to determine what other genes might be responsible for tumor development [6]. 

Symptoms of ENB vary depending on the location of the tumor and the extent of the disease and the stage at presentation. The most common symptoms are unilateral nasal obstruction, nasal bleeding, headache, facial pain, and a decreased sense of smell. Extension of the tumor to the eyes or the cranial cavity and the nasal cavity can lead to symptoms related to these areas. Serous otitis media can develop due to the obstruction of the eustachian tube. Sinonasal symptoms are the most common in this condition, and they mimic the symptoms of inflammatory disease in the area and could be confused as such, leading to misdiagnosis delaying effective treatment [7].

It is worth noting that 20% of patients who present with ENB will have neck metastasis so it is important for proper diagnosis to take a good history and perform a focused physical exam with an emphasis on the neck examination. Nasal endoscopy is then performed to locate the tumor, stage it and obtain a biopsy [8].

When suspecting ENB the initial test that should be performed is a high-resolution computerized tomography (CT) scan. This allows for superior delineation of bony structures and whether they are intact or broken. It is of critical importance to observe the orbit, skull base, septum, and palate. High-resolution CT is the imaging modality used initially. Each patient with suspected ENB needs to undergo a CT scan and a magnetic resonance imaging (MRI) scan as part of the standard imaging procedure. This is critical to evaluate lesion extent, and involvement of the surrounding structures like the orbit, skull base, dura and brain parenchyma. CT of the neck, chest, and abdomen is the next step to evaluate the possibility of metastasis. Positron emission tomography (PET) scan is an option to investigate distant metastasis as well [9-10].

Several staging and grading systems have been developed to assess ENB. Hyams grading system is used for prognosis and grading while the Kadish system is for staging the disease. These are the most widely used systems in modern day literature. The difficulty in validating any staging system in the case of ENB is due to the low incidence of disease among other variables [11-12].

## Review

Methods

This is a literature summary designed to report relevant center experience concisely and clearly to qualitatively report findings on which of the currently used protocols provide the lowest mortality and morbidity in clinical trials conducted from 1990 to January 2019. The objective of this study is to report the treatment option that has the best progression-free survival and overall survival. All clinical trials published since 1990 until January 2019 were selected. Studies were required to be written in English and include a sample of adult males and females. Databases used include PubMed, Medline, the Cochrane Collaboration. Keywords were: “Esthesioneuroblastoma”, “ENB”, “Olfactory Neuroblastoma”, Nose neoplasm”, skull base neoplasm”, “radiation”, and “resection”. The search yielded 684 articles. Limitations used were: published after 1990, English language, and clinical trials. Results were excluded if written before 1990, written in languages other than English and if the sample under study is not adult males or females. Studies that did not address the question directly were also excluded.

The search yielded seventeen papers that were included in this review. The criteria to accept papers was based on the clear reporting of outcomes post-intervention. Fourteen single-center experiences, one systematic review, two nationwide multiple center retrospective reviews were included. Studies that analyzed the same sample and reported the same prognostic variables were excluded from the summary but added in the discussion as relevant. This exclusion is to limit redundancy in analyzing findings and to avoid exaggerating results. Data reported in this review are summarized in Table 1. 

**Table 1:** Summary of included studies AR: Adjuvant radiation, CFR: craniofacial resection, DFS: Disease-free survival, ECR: Expanded-endoscopic, endonasal approach, EEA: Endoscopic endonasal approach, ENB: Estheisioneuroblastoma, F: Female, M: Male, n: Sample size, NAR: Neoadjuvant radiation therapy, NCDB: National Cancer Database, NED: No evidence of disease, ONB: olfactory neuroblastoma, OS: Overall survival, Pts: Patients, RT: Radiation therapy, TFR: transfacial resection without craniotomy.

Study ID	Design	Sample	Staging/metastasis	Treatment option	Follow up	survival	commentary
Ow, et al., 2013 [13]	Retrospective review 1992-2007	N=70 ENB patients	77% were T-3 or T-4 38% were modified Kadesh B or C	90% received surgical resection 66% received post-operative radiation or chemotherapy	Median follow up: 91.4 months (7.6 years)	48% developed recurrent disease. Median time to recurrence: 6.9 years.	Surgery alone: 87.9 months survival Surgery + chemo/ radiation: 218.5 months survival.
Herr et al., 2014 [14]	Retrospective chart review 1997-2013	N=22 ENB patients	Kadesh stage B: 10 patients, stage C: 12 patients 27% developed regional metastasis	All received CFR+ radiation therapy With/without chemo	Average follow up: 73 months	5 years disease free and overall survival: 86.4% and 95.2% respectively	Photon beam radiation showed lower toxicity than other radiation options.
Tajudeen et al., 2014 [15]	Retrospective review 2002-2013	N=41 (36 included) ENB patients UCLA medical center	Kadesh A: 2 Kadesh B: 15 Kadesh C: 20 Kasesh D: 4	CFR: 8 pts. TFR: 20 pts. ECR: 8 pts.	Mean follow up: 31.5 months	5 years recurrence free and overall survival: 54% and 82% respectively	All methods showed comparable outcomes in survival
Yin et al., 2015 [16]	Retrospective review of center records 1979-2014	N=111 patients with ENB	Stage A: 1 pts. Stage B: 23 pts. Stage C: 87 pts. N+: 27 pts.	Surgery + RT ± Chemo: 51 pts. Preoperative RT + surgery + Chemo: 11 pts. RT + chemo: 46 pts. Surgery ± chemo: 3 pts.	Mean follow up 5 years	Stage A: 19 years Stage B: (OS: 81%, DFS: 71%) Stage C: (OS: 71%, DFS: 49%)8	preoperative RT + surgery indicated best survival.
Chowdhury et al., 2015 [17]	A 24 years retrospective review at university of Kansas medical center	N= 44 ENB patients (38 were included in the review)	Not recorded	All patients underwent surgical resection. 79% received post-operative radiation as well. 60% of whom received radiation 2 months late.	Mean follow up was 81 months	A 10 weeks delay of radiation therapy post-surgical resection increased metastasis risk by 50%.	Delayed radiation could potentially increase risk of metastasis
Sharett et al., 2015 [18]	Retrospective review of patient records 1970-2013	N=75 pts. (surgery only patients were excluded from review)	Kadesh stage > C: 77%	All patients received radiation therapy. 88% post operatively. 12% preoperatively. 26.6% received chemotherapy	Median follow up: 105 months	5 and 10 years OS rates were 87% and 74% respectively. 93% and 81% were free from distant metastasis at 5 and 10 years follow up respectively.	Combined therapy provides the best predictor of survival and disease free time. Exclusion of patients who received surgery only is problematic and requires explanation by the authors of what happened to them at follow up.
Lapierre et al., 2016 [19]	Retrospective review of patient data at Lyon Sud University Hospital (France) 1993-2015	N= 10 pts.	Kadesh stage C: 90%	Surgical resection (9 pts) + adjuvant radiation (7 pts) or chemotherapy (2 pts)	Median follow up was 136 months	Ten-year overall survival was 90%. Five- and ten-year progression-free survival were 70% and 50%	None of the patients received nodal irradiation 50% of patients had disease recurrence
Agarwal et al., 2017 [20]	Retrospective review at Mayo clinic, Rochester	N=109 pts (only 45 met the inclusion criteria)	Kadesh stage B or C (selection criteria)	Surgical resection + radiation therapy AR: 22 pts. Surgical resection with no adjuvant radiation NAR: 9 pts.	Mean follow-up was 103.4 ± 60.3 months	AR: 9 dead, 7 secondary to ENB at last follow up. NED: 8 NAR: 5 developed recurrence at mean 50.8+/-50.9 months. Received radiation. All were alive at last follow up. NED: 6	Little toxicity incurred due to immediate radiation. Patients undergoing delayed radiation developed recurrence at time of salvage surgery were all alive at the last follow up.
Xiong et al., 2017 [21]	Retrospective review of patient data in a Chinese center 1981-2015	N=187	Kadesh A: 23 Kadesh B: 48 Kadesh C: 113 Unknown stage: 3	Surgery + RT +chemo:117 Surgery + RT: 35 Surgery alone: 32 Palliative care only: 3	Mean follow up was 3 years.	Surgery and combined radiotherapy with or without chemotherapy led to better OS and DFS than other treatment modes	Surgery and combined therapy is the optimal modality of treatment for patients with ENB. Follow up is short compared to the literature Not receiving combined modality was an independent factor for poor OS and DFS.
Nakagawa et al., 2017 [22] (Multicenter, Japan)	Retrospective review of patient records from 10 centers in Japan between 2008-2016	N= 22 10 M 12 F	Dulguerov staging at presentation was: T1: 6 patients T2: 9 patients T3:5 patients T4:2 patients	unilateral resection via EEA was performed in 12 patients bilateral resection via EEA was done in 10 patients Post-operative radiotherapy was done in 20 patients	Mean follow up was 44 months. All patients were alive at last follow up.	Local recurrence observed in 1 T2 patient 12 months post bilateral resection	Multilayer resection with EEA is a safe method to treat ENB. Surgery + radiotherapy provides an excellent combination for the treatment.
Lui et al., 2017 [23]	Retrospective review of medical records at a single center from 1986-2016	N= 42	Kadesh A: 7 Kadesh: B: 8 Kadesh C: 27	Surgery + RT: 33 pts Surgery alone: 6 pts Preoperative rt + surgery: 2pts RT: 1pts	Median follow up: 87 months	Kaplan-Meier 5 and 10 years overall survival: 83% and 72% respectively. Kadesh C is worse than Kadesh A/b combined: 57% vs 88% Kaplan-Meier 10 years overall survival	Surgery and radiation therapy provide the most favorable outcomes even with locally advanced disease.
Palejwala et al., 2017 [24]	Retrospective review of medical records at single center 2006-2016	N= 8	Kadesh A: 4 Kadesh C, D: 4	Kadesh A: endoscopic approach Kadesh C, D: craniofacial approach All patients received RT post-surgery.	Average follow up was 60.4 months	Average progression free interval was 57 months. Overall survival was 88% at the end of the study.	Complications occurred in high Kadesh stages only.
Carey et al., 2017 [25]	Retrospective review of NCDB database for ENB patients in the united states	N=1225 (1118 were included in the analysis)	Kadesh (n): A: 225 B: 167 C: 597 D: 31 Unknown: 98	Surgery: 242 Radiation: 19 Chemo: 22 Surgery+ radiation: 383 Surgery+ chemo+ radiation: 182 Surgery+ chemo: 19 Radiation then surgery: 12 Radiation before and after surgery: 3 Radiation before and after surgery + chemo: 2	Multivariate analysis of NCDB. Follow up time not specified.	the 5-year overall survival was 76.2%	surgery followed by radiation without chemotherapy had improved all-cause mortality. Surgery followed by chemotherapy has worse overall survival for Kadesh C pts.
Gallia et al., 2018 [26]	Retrospective chart review of 20 patients with ONB between 2006 and 2017	N=20	Not reported.	Surgery: 20 Surgery + radiotherapy: 19 Surgery + radiotherapy + post operative chemotherapy: 5	Mean follow up: 5 years	At 5 years 19 pts were alive. 1 pt died from unrelated illness. Overall survival: 92.2% Disease specific survival: 100% Recurrence free survival: 92.9%	The findings support the continued use of endoscopic procedures to treat ONB.

ENB is a rare condition that affects the nasal cavity. Due to the rarity of the disease and different treatment modalities in different centers, generalizations about single center experiences is difficult. Moreover, there is heterogeneity that comes with the findings that must be kept into consideration while evaluating the results. In this review, one theme can be ascertained very quickly, which is the clear trend towards far superior overall survivability and disease-free survival with multimodal interventions; namely surgical resection followed by radiation therapy [18-23]. In all studies included in this review, this finding has been consistent regardless of the resection type. This finding becomes more apparent with higher disease stages as surgery followed by radiation therapy showed longer disease-free progression in Kadesh stage C in particular [25-28].

It is worth noting that in two studies delayed administration of radiation post-surgical resection was associated with a higher probability of disease recurrence and metastasis suggesting the necessity of fast and aggressive introduction of adjuvant radiation therapy early after surgical resection [15,19,21]. When comparing two groups, the first receiving radiation therapy post-surgery and the other group receiving it six weeks to two months’ post-surgery, the authors found that although the patients receiving the radiation therapy immediately post-surgery had slightly higher levels of toxicity than the patients who did not receive it promptly, they did better than the delayed group in the disease-free time and time to recurrence with metastasis [20].

One very important aspect we think needs further research is whether the introduction of nasal endoscopic surgical techniques as compared to conventional surgical techniques for ENB treatment accelerated the healing process opening the door for a timely use of adjuvant radiation therapy which could lead to an even better outcome in terms of disease-free survival. The current literature does not show any significant difference between surgical techniques on their own in terms of outcome [15]. However, it would be worth investigating whether techniques with better healing time coupled with the rapid introduction of radiation therapy would be superior to other techniques. 

As for using radiation and when to use it pre or post-surgical resection, most studies in this review used radiation post-surgical resection, however, the reasoning for this choice is not clear. It is true that this is the standard practice by ENT and neurosurgical teams, however, the basis for this choice is not challenged by other options thus far. An example of a challenging outcome would be Yin et al. (2016) who used radiation therapy before surgical resection and found the results to be superior to using it after surgery in terms of disease-free survival [16]. This study given its sample size has a significant weight in begging the question of when radiation needs to be done pre or post-surgery for the best overall and progression-free survival.

## Conclusions

ENB is a rare olfactory neoplasm that requires careful evaluation and prompt diagnosis. Aggressive treatment is necessary to improve patient disease-free and overall survival. This review concludes that surgical resection followed by radiation therapy provides the best disease-free survival and overall survival. The role of chemotherapy post surgery is potentially harmful to disease-free survival and overall survival and thus should be discouraged until further research is conducted to ascertain the degree of benefit and harm to patients. 
